# Temperament profile and its association with the vulnerability to smartphone addiction of medical students in Indonesia

**DOI:** 10.1371/journal.pone.0212244

**Published:** 2019-07-11

**Authors:** Enjeline Hanafi, Kristiana Siste, Tjhin Wiguna, Irmia Kusumadewi, Martina Wiwie Nasrun

**Affiliations:** Department of Psychiatry, Faculty of Medicine, Universitas Indonesia, DKI Jakarta, Indonesia; University of Auckland, NEW ZEALAND

## Abstract

Two dimensions of temperament, namely, (high levels of) novelty seeking and (low levels of) harm avoidance are related to substance addictions. However, their implications for smartphone addiction remain unexplored. Medical students are heavy smartphone users. Accordingly, screening for the risk of smartphone addiction based on individual differences in temperament can facilitate the identification of the best possible prevention strategy. Therefore, the present study aimed to examine the relationship between temperament and the vulnerability to smartphone addiction among medical students in Jakarta, Indonesia. The research study adopted a cross-sectional research design and used a simple random sampling technique. The Indonesian versions of the Temperament and Character Inventory and the Smartphone Addiction Scale were used to measure the study variables. Logistic regression analysis was conducted to examine the relationships between demographic factors, patterns of smartphone use, temperament, and vulnerability to smartphone addiction. A majority of the 185 participants were found to have the following temperament profile: low levels of novelty seeking and high levels of reward dependence and harm avoidance. The average duration of daily smartphone use was 7.83 hours (SD = 4.03) and the age at first smartphone use was 7.62 years (SD = 2.60). The respondents used smartphone to communicate with other people and access social media. A high level of harm avoidance was significantly associated with the risk of smartphone addiction (Odds Ratio [OR] = 2.04, 95% Confidence Interval [CI] = 1.12, 3.70). The findings suggest that smartphone addiction is comparable to other addictive behaviors. Further, harm avoidance increases the risk of smartphone addiction. Therefore, the risk of smartphone addiction among medical students must be ascertained based on their temperament profiles.

## Introduction

Smartphones, which are mobile phones that provide integrated communication and computation services, play an important role in people’s lives worldwide [1,2]. According to a study that was conducted by the Pew Research Center, smartphone ownership is strongly related to internet connectivity [3]. The number of smartphone owners and internet users has drastically increased across the past few years. For example, in developing countries, smartphone ownership increased from 21% in 2013 to 37% in 2015 [3]. This dramatic increase in the number of smartphone users raises questions about its risks. In general, smartphone use is associated with motor vehicle accidents, academic problems, and health issues [4]. Frequent use of smartphones increases the prevalence of sleep disorders, depression, and anxiety [5]. Evidently, smartphone overuse has a significant impact on psychological well-being.

Extremely high levels of smartphone use can lead to dependence or addiction. According to one study, 46% of smartphone users reported that they cannot live without smartphones [3]. Another study showed that, on average, university students check their phones 60 times a day and spend more than 4 hours on their phones. The incumbent fear of separation from one’s smartphone has been termed as “nomophobia” [6]. When such a separation occurs, withdrawal-like psychological symptoms (such as restlessness) and physiological symptoms emerge [7,8]. Some individuals experience phantom vibration and ringing (i.e., the perception that one’s phone is vibrating or ringing despite a lack of corresponding stimuli; 8). This phenomenon is not the same as compensation; indeed, its objective entails neither an escape from problems or responsibilities nor an avoidance of negative emotions [9]. Nevertheless, the symptoms of smartphone addiction and withdrawal can adversely impair functioning, such as reported from a study by Aguilera-Manrique et al [10]. However, there has been no attempt to estimate the prevalence of smartphone addiction in Indonesia. Although some studies have been conducted in other Asian countries [4, 11], they have reported high prevalence rates such as 37.9% (China) and 14.2% (South Korea). These rates are substantially higher than those that have been reported for European countries (12.5% to 21.5%; [12]).

Early detection, which is the first step toward the mitigation of the consequences of smartphone addiction, should take individual risk factors into consideration; temperament is one such risk factor [13]. There has been no past attempt to explore the association between temperament and smartphone addiction. Temperament refers to the patterns of emotions, moods, and sensory responses that characterize an individual. It is free from the influence of values and norms, and it is associated with specific brain systems that regulate negative emotions and behaviors [14]. According to Cloninger, temperament has three dimensions: *novelty seeking* (NS), *reward dependence* (RD), and *harm avoidance* (HA) [15]. Novelty seeking is a behavioral activation in response to novelty and signals of reward or relief of punishment; reward dependence is a behavioral inhibition that occurred in response to signals of punishment or nonreward; and harm avoidance is a behavior that was previously rewarded was later maintained for a while without continued reinforcement [14]. An individual’s temperament profile can provide the reasons that underlie their thoughts, perceptions, and behaviors. Ko et al. conducted a study on the role of temperament in behavioral addiction [16]. They found that individuals who had been diagnosed with internet addiction had high levels of NS and HA, and low levels of RD. However, there has been no past attempt to examine the role of temperament in smartphone addiction. Such investigations are necessary because the risk factors for internet addiction may not be generalizable to smartphone addiction.

Eley et al., found that Australian medical students tend to have high levels of HA, which are comparable to those that have been observed among individuals who have been diagnosed with internet addiction [17,18]. It is reasonable to speculate that similar temperamental profiles may be characteristic of medical students in Jakarta because they use smartphones and other similar gadgets to keep themselves abreast of recent developments in the field of medicine [19]. Additionally, medical students undergo long durations of study and set high expectations for themselves [20]. Consequently, smartphones that are initially used for academic purposes may eventually serve as a means of escape from challenging situations. If such behavior patterns are neglected, they may adversely affect academic achievement, daily life, and future achievement [21]. Screening for high-risk students can facilitate the prevention of smartphone addiction among students; however, it is not feasible to provide this service to all students. Accordingly, intervention strategies that aim to prevent or manage smartphone condition must identify at-risk individuals based on a survey of temperament profiles. Therefore, the study aimed to examine the temperament profiles of medical students in Jakarta and their association with the vulnerability to smartphone addiction.

## Methods

The present study adopted a cross-sectional research design, and it was conducted across several medical institutions in Jakarta. Jakarta is the capital city of Indonesia, and it is the third most populous country in the world. The students who belonged to the aforementioned medical institutions in Jakarta originated from all over Indonesia. Data collection was undertaken from January to April 2018.

### Participants

The sample for the present study consisted of preclinical medical students in Jakarta who fulfilled the inclusion and exclusion criteria. The target sample size was estimated using the sample size formula that is to be used for cross-sectional studies and the following values: Zα = 1.96, absolute precision = 0.05, and the prevalence of smartphone addiction = 14% [11]. The participants were selected using simple random sampling, and randomization was conducted using the software for analysis (Statistical Package for Social Sciences Windows or SPSS). The study participants were recruited during a plenary session that was conducted in each medical faculty. The attendees were presented with a description about smartphone addiction and the aim of the present study; those who were willing to participate in the study were required to sign an informed consent form.

The sample inclusion criteria were as follows: preclinical medical students who were in the fifth semester of their course, had completed the demographic form and the two questionnaires that were used in the study, and had provided informed consent by signing the corresponding form. Participants were excluded if they were not fluent in Indonesian because limited language skills can impede their understanding of the contents of the instruments that were used in the study.

### Measurements

#### The Indonesian version of the Smartphone Addiction Scale (SAS)

Vulnerability to smartphone addiction was measured using the self-report SAS. This instrument consists of 33 questions, and responses are recorded on a Likert-scale that ranges from 1 to 6. The scale consists of 6 subscales: cyberspace-oriented relationship, daily life disruption, primary needs, overuse, positive anticipation, and withdrawal [11]. This instrument has been found to be both reliable (Cronbach’s α = 0.967) and valid. Additionally, the Indonesian translation of the SAS has also been validated [22]. The Indonesian version of the SAS consists of 21 questions, and its item-total correlations range from 0.282 to 0.802 (Cronbach’s α = 0.890). The SAS yields a numerical score, and higher scores signify a greater vulnerability to smartphone addiction. Since the SAS does not specify thresholds that are indicative of the risk for smartphone addiction, high and low risk for smartphone addiction were defined in terms of the scores that lay above or below the average SAS score that was obtained by the study sample, respectively.

#### The Indonesian version of the modified Temperament and Character Inventory (TCI)

The Indonesian version of the TCI was used to measure temperament [23]. This version had been validated using confirmatory factor analysis. It consists of 23 questions that assess temperament (9, 6, and 8 items measure novelty seeking, reward dependence, and harm avoidance, respectively) and 16 questions that assess character (10 and 6 items measure self-directedness and self-transcendence, respectively). The split-half reliability of this instrument was found to be 52%. In the present study, we used only the questions that assess temperament.

Accordingly, the 23 self-report items, which measure temperament, entail two response options: “Yes” (2 points) and “No” (1 point). The item responses are to be scored in accordance with the answer key. The composite scale score can be obtained by summing the scores that have been computed for each of the three dimensions; accordingly, higher composite scores are indicative of higher scores on the three dimensions (i.e., harm avoidance, novelty seeking, and reward dependence). In the present study, the average scores that were obtained by the sample on the three dimensions served as the thresholds based on which the participants were classified into “low” and “high” groups.

### Ethical approval

The present study was approved by the local Ethics Committee (No. 150/UN2.F1/ETIK/2017). The confidentiality of the participant responses was ensured. The medical institutions were provided with the overall findings of the present study but not the personal information of any of the study participants.

### Statistical analysis

The data that were collected were subjected to statistical analyses, which were executed using version 25 of Statistical Package for Social Sciences for Windows (SPSS, IBM, USA). With regard to the demographic factors, proportions were used for categorical variables, and means and standard deviations were computed for continuous variables. Logistic regression analysis was used to examine the relationship between demographic factors, smartphone use, temperament, and the vulnerability to smartphone addiction.

## Results

The sample that was used in the present cross-sectional study, which consisted of 300 medical students who were in the fifth semesters of their medical courses, were recruited from three medical institutions. However, the forms of only 258 participants fulfilled the study criteria. Subsequently, 185 participants were randomly selected from this pool.

### Demographic characteristics of the participants

The mean age of participants was 20.39 years (SD = 1.14), and a majority of the participants were women (66.50%); none of the participants were married (Table 1).

**Table 1:** Demographic characteristics of the participants.

Demographic characteristic	Mean (SD)	95% CI		n	%
		Lower limit	Upper limit		
**Age (years)**	20.39 (1.14)	20.23	20.56		
**Marital status**					
Married				0	0
Not married				185	100
**Gender**					
Man				62	33.50
Woman				123	66.50

Note. SD = standard deviation; CI = confidence interval.

### Smartphone use among participants

The participants primarily used their smartphones to communicate with others (41.10%) and access social media (29.70%). The mean duration of daily smartphone use was 7.83 hours (SD = 4.03); 68.60% of the subjects used their smartphones for 6 hours or more. The mean age at which participants first used a smartphone was 7.62 years (SD = 2.60; Table 2).

**Table 2:** Smartphone use among participants.

Variable	Mean (SD)	95% CI		n	%
		Lower limit	Upper limit		
**Age at first smartphone use (years)**	7.62 (2.60)	7.24	8.00		
**Duration of daily smartphone use (hours)**	7.83 (4.03)	7.24	8.41		
≥ 6 hours				127	68.60
< 6 hours				58	31.40
**Primary purpose of smartphone use**					
Communication				76	41.10
Browsing the internet				26	14.10
Playing games				12	6.50
Social media				55	29.70
Entertainment				16	8.60

Note. SD = standard deviation; CI = confidence interval. Entertainment consisted of streaming film, listening to music, reading novel or ebook.

### The purpose of smartphone use: Gender comparisons

Men used smartphones to communicate with others (35.50%), access social media (24.20%), and play games (19.40%). On the other hand, women used smartphones to communicate with others (43.10%), access social media (33.30%), and browse the internet (13.80%). There was a significant gender difference in the use of smartphones for the purpose of playing games (χ^2^ = 21.69, p < 0.001; Table 3).

**Table 3:** Gender differences in the purposes of smartphone use.

Purpose of smartphone use	Gender		p
	Men	Women	
Communication	23 (37.10%)	53 (43.10%)	0.53
Browsing the internet	10 (16.10%)	16 (13.00%)	0.66
Playing games	10 (16.10%)	2 (1.60%)	<0.001*
Social media	14 (22.60%)	41 (33.30%)	0.17
Entertainment	5 (8.10%)	11 (8.90%)	1.00

Note.

*p < 0.05.

### The temperament profile of the participants

The mean scores for the three dimensions of temperament, namely, NS, RD, and HA were 11.56 (SD = 1.89), 9.36 (SD = 1.69), and 11.74 (SD = 1.88), respectively. Those who obtained scores that lay above these mean scores were classified as “high” with regard to the respective dimension of temperament. More than 50% of the participants were classified as “low” with regard to NS and as “high” with regard to RD and HA (Table 4).

**Table 4:** The temperament profile of the study participants.

Temperament dimension	Mean (SD)	95% CI		n	%
		Lower limit	Upper limit		
**Novelty seeking**	11.67 (1.80)	11.41	11.93		
High				89	48.1
Low				96	51.9
**Reward dependence**	9.44 (1.66)	9.20	9.68		
High				98	53.0
Low				87	47.0
**Harm avoidance**	11.65 (1.91)	11.37	11.93		
High				98	53.0
Low				87	47.0

Note. SD = standard deviation; CI = confidence interval.

### Logistic regression: The relationship between smartphone use and temperament

The average SAS score that was obtained by the study participants was 77.90 (SD = 11.73). Approximately 51% of the students obtained scores that lay above the average SAS score. Multivariate logistic regression analysis was used to examine the relationships between the following three variables which had p<0.25 in the univariate logistic regression (Table 5): 6 or more hours of daily smartphone use, smartphone use for the purpose of entertainment, and high HA score (Table 6). The results of the Hosmer-Lemeshow test indicated that this model was acceptable (p = 0.72).

**Table 5:** Results of the univariate regression analysis.

Variable	B	p	Odds ratio	95% CI	
				Lower limit	Upper limit
≥ 6 hours of smartphone use per day	0.62	0.07	1.86	0.97	3.58
Communication	0.22	0.83	1.25	0.65	2.38
Browsing	-0.21	0.45	0.81	0.32	2.03
Games	-0.79	0.42	0.45	0.12	1.67
Social media	-0.22	0.71	0.80	0.39	1.65
Entertainment	0.85	0.07	2.35	0.67	8.25
High NS	-0.04	0.74	0.96	0.52	1.75
High RD	-0.24	0.79	0.79	0.42	1.46
High HA	0.77	0.02	2.15	1.17	3.98
Constant	-0.48	0.27	0.62		

Note. CI = confidence interval; Hosmer-Lemeshow test χ^2^ = 3.31, df = 7, p = 0.86; Nagelkerke’s R^2^ = 0.10;

*p<0.05.

**Table 6:** Results of the multivariate logistic regression analysis.

Variable	B	p	Odds ratio	95% CI	
				Lower limit	Upper limit
≥ 6 hours of daily smartphone use	0.59	0.07	1.80	0.95	3.43
Entertainment	0.99	0.10	2.71	0.82	8.98
High HA	0.71	0.02*	2.04	1.12	3.70
Constant	-0.74	0.02	0.48		

Note. CI = confidence interval; Hosmer-Lemeshow test: χ^2^ = 2.07, df = 4, p = 0.72; Nagelkerke’s R^2^ = 0.09;

*p < 0.05.

The results of the aforementioned multivariate analysis revealed that high HA was a statistically significant predictor (Odds ratio [OR] = 2.04; 95% Confidence interval [CI] = 1.12, 3.70) and that the other two variables, namely, the duration of daily smartphone use (OR = 1.804; 95% CI = 0.95, 3.43) and the use of smartphone for entertainment purposes (OR = 2.71; 95% CI = 0.82, 8.98) were confounding factors.

## Discussion

The present study is the first to examine the relationship between temperament and the risk for smartphone addiction among young adults. In addition to temperament, the present study also explored other factors such as age, marital status, gender, duration of smartphone use, and age at first smartphone use.

The mean duration of smartphone use that emerged in this study (i.e., 8 hours per day) is higher than those that have been reported in other studies. Specifically, others have reported a mean daily duration of 3.7 hours [24] or less than 1 hour per day [25]. This difference may be attributable to the differences in sample characteristics between past studies and the present study. For example, Lin et al. conducted their study using a sample of engineering students whose smartphone use tends to be lower than that of medical students [24]. Similarly, in Cho and Lee’s study, the sample consisted of children who are subjected to parental supervision [25]. In the present study, we did not find a significant relationship between the duration of smartphone use and the risk of smartphone addiction. However, past studies found that such a relationship does exist [26,27]. One of the diagnostic criteria for addiction is tolerance. A person who is addicted to smartphones will have to engage in longer durations of smartphone use to derive the desired level of satisfaction than their non-addicted counterparts.

The age at first smartphone use is an important factor that plays a role in the development of addiction. In the present study, the age at first smartphone use was relatively low (i.e., 7.62 years). Ching et al. proposed that a younger age at first smartphone use is related to a higher risk for addiction [28]. Adolescents are vulnerable to addiction because their underdeveloped prefrontal cortex precludes them from adequately judging the causal implications of their actions [29,30]. Zhitomirsky-Geffet and Blau conducted a transgenerational study and found that adolescents who are first exposed to mobile phones during their teenage years are more likely to develop smartphone addiction because their personalities are more malleable during this developmental period [9].

Smartphone use can be motivated by a variety of reasons. For example, smartphone use gratifies the basic need for communication (e.g., calls, short text messages); however, in recent times, people use their phones to access social media, browse the internet (as well as shop online), and play games for long periods of time [31]. In other words, the purpose of smartphone use has changed from communication to entertainment [32,33].

The search for entertainment is an important theme because it reflects a person’s coping mechanism [34,35]. Accordingly, Nayak proposed that smartphone use among students is related to a wish to escape from reality, even if only temporarily [36]. Such avoidant coping strategies are often associated with health problems and depression [37]. Coping mechanisms are related to not only psychopathology but also temperament. The temperament that is most closely related to avoidant coping mechanisms is (high levels of) HA [38].

The participants of the present study constituted a homogenous group because they all belonged to the same stage of their medical course. Eley et al. found that medical students are a relatively homogenous group of individuals who tend to be in their early 20s, have a good educational background, and belong to upper-middle socioeconomic class [16]. In the present study, we found that medical students are homogeneous with regard to marital status. Thus, the evident homogeneity of the study sample may influence the other results that emerged in the present study (e.g., those that pertain to temperament type and the type of smartphone activity).

In the present study, a majority of the participants were women. Similar to past findings, temperament differed across the two genders. The higher HA scores that were obtained by women may be attributable to gender differences in the monoamine system and hormonal composition. The high HA scores that were obtained by women suggests that they have more active serotonin and noradrenaline components. Further, estrogen, which is sensitive to the HPA axis, may render women more vulnerable to anxiety [39]. Additionally, women acquire socialization skills at a rate that is faster than that of men [15].

Women’s tendency to socialize to a greater extent concurs with the present finding that women were more likely to use smartphones to access social media than men. Women’s tendency to use smartphones to access social media has been attributed to their tendency to have both wider social networks as well as deeper relationships, when compared to men who tend to have only a wider social network [1,19]. Smartphones may allow women who have high levels of HA to socialize more conveniently.

In the present study, men used smartphones to play online and offline games. When compared to women, men are more likely to be active gamers because they tend to be more task-oriented [40]. A majority of gamers exhibit this trait; consequently, such activities are more interesting to men. Furthermore, games serve as a means by which men channel their aggression [33]. Easterners, especially Asians, tend to obtain higher mean HA scores than Americans and Europeans [41]. Accordingly, aggression may be channelized by engaging in non-threatening activities (e.g., playing video games; [42]) because Asian communities tend to be less individualistic and more collectivistic [41]. In addition to the role of culture, the expression of aggression in cyberspace is associated with biological factors (e.g., brain structure, hormones, neurotransmitters) that influence social cognition. Smartphone use for the purposes of accessing social media and playing games may be beneficial up to a certain threshold; however, as noted in an earlier instance, overuse is associated with avoidant coping strategies.

A majority of the participants in the present study had high HA. In addition to gender, this temperament profile can be attributed to the fact that the study sample was recruited from medical institutions. Similarly, Eley et al. observed concurrent trends across two different studies that were conducted among medical students in Australia: low NS, high RD, and high HA [18]. Lee et al. concluded that a combination of high levels of HA, cooperativeness, and self-directedness can help medical students develop into independent, reliable, warm, empathic, and friendly doctors [20]. However, such types of individuals are also vulnerable to burnout, anxiety, and depression [17,18,43].

In the present study, a high risk for smartphone addiction was approximately twice as high for those with high rather than low levels of HA. This finding is similar to those of other studies on internet addiction [13,44,45]. A person with high levels of HA is more likely to be addicted to smartphones when compared to students with high levels of NS. This is attributable to the low persistence of NS, which may cause an individual to feel bored more easily and impulsively shift to other activities.

In the present study, the duration of smartphone use that is 6 hours or more was not significantly associated with smartphone addiction. However, it is an important confounding factor that renders an individual vulnerable to smartphone addiction. Other studies have also found that the duration of smartphone use is related to smartphone addiction [12,21,26]. This duration is indicative of an individual’s priorities and the extent to which smartphone use interferes with their daily functioning. Consequently, a change in self-related priorities is one of the diagnostic criteria for addiction in both the Diagnostic and Statistical Manual of Mental Disorders-5 as well as the International Classification of Diseases -11 [46,47].

Smartphone use for the purpose of entertainment was positively related to vulnerability to smartphone addiction. Two studies have reported a significant relationship between smartphone use for entertainment purposes and smartphone addiction [21,48]. Further, Boumosleh and Jaalouk observed that the use of smartphones to search for entertainment is related to the negative coping mechanisms that are used to alleviate depression and anxiety [21]. Thus, the more frequently a person uses such coping mechanisms, the more frequently he or she is likely to use smartphones for entertainment purposes. Although we speculate that a longer duration of smartphone use may increase the risk for smartphone addiction, which eventually leads to a vicious cycle, such a contention was not tested in the present study; therefore, further research is required to examine the directionality of the emergent relationships.

In general, the present findings suggest that smartphone addiction is similar to other addictive behaviors such as pathological gambling, internet addiction, and online gaming addiction [49]. Even though all the factors that lead to smartphone addiction were not examined in the present study, the findings suggest that many factors such as educational background and social and personal factors play a role in the vulnerability to smartphone addiction [1,44,45,50].

The present study has several limitations. First, the present study was conducted using a small sample of medical students in Jakarta. Additionally, we did not compare our sample to a non-medical sample. These limitations restrict the generalizability of the present findings. Second, the present study did not take into account the types of application that can be used through smartphones; indeed, there are a few multifunctional applications. Additionally, the type of games that participants played (e.g., online vs. offline games; genre: action, adventure, role-playing games, and puzzles) was not examined. Therefore, future studies that examine the specific types of applications and games that are related to smartphone use can provide a more nuanced interpretation of the present findings. Third, there is a lack of consensus about the cutoff scores for the SAS based on which participants are to be classified into the high- and low-risk categories. Accordingly, a replication of the present study that utilizes different cutoff scores will yield different results. Fourth, the present study examined temperament as a risk factor for smartphone addiction. However, there are other unexplored factors that influence both temperament and smartphone addiction (e.g., genetics, parenting, character, self-regulation, self-image, comorbid mental disorders, and peer pressure). Finally, individual differences in the impact of smartphone use was not examined in the present study.

The findings of the present study suggest that HA is a risk factor for smartphone addiction. Specifically, a higher tendency for HA is associated with a higher risk for smartphone addiction. Accordingly, medical students’ risk of developing smartphone addiction must be ascertained based on their temperament profiles. Early exposure to the literature on behavioral addiction as a part of the medical program can serve as a preventive measure against addiction. Further, future research must examine other biological, psychological, and social factors that influence a person’s vulnerability to smartphone addiction. Such studies must also examine psychopathological variables as potential risk factors for smartphone addiction.
